# The role of sexual self-esteem, sexual desire, and sexual assertiveness in the female sexual function

**DOI:** 10.1186/s41155-024-00303-4

**Published:** 2024-06-11

**Authors:** Shokoufeh Roshan Chesli, Zahra Bostani Khalesi, Sara Shirzad Chenari

**Affiliations:** Social Determinants of Health Research Center, Guilan University of Medical Sciences, Rasht, Iran

**Keywords:** Sexual assertiveness, Sexual desire, Sexual self-esteem, Sexual function

## Abstract

**Background:**

Sexual function plays a very important role in the sexual health of people, and the determination of their related factors reflects the importance of paying attention to sexual function in the cultural context.

**Objective:**

The present study aimed to the role of sexual self-esteem, sexual desire, and sexual assertiveness in the female sexual function.

**Methods:**

In this descriptive-analytical cross-sectional study, 592 married women of reproductive age referring to comprehensive health centers in Rasht city (North of Iran) and eligible for the inclusion criteria were selected by cluster random sampling. The data collection tools were demographic information form, Halbert’s Sexual Rights Questionnaires, Women’s Sexual Self-Esteem (short form), Halbert’s Sexual Desire, and Female Sexual Function Index. Data analysis was done with descriptive and inferential statistical tests at a significant level (*p* < 0.05).

**Results:**

The mean and standard deviation of sexual assertiveness, sexual desire, and sexual self-esteem scores were 56.79 ± 18.24, 49.12 ± 26.04, and 98.52 ± 6.11, respectively. Sexual assertiveness (*p* < 0.01, *r* = 0.13), sexual desire (*p* < 0.001, *r* = 0.178), sexual self-esteem (*p* < 0.01, *r* = 0.34) of the participants with the total score, and all areas of female sexual function had a significant positive correlation.

**Conclusion:**

Based on the findings, there is a direct and statistically significant relationship between sexual assertiveness, sexual desire, the adaptability of sexual self-esteem, and family income with sexual function in participants. However, the unemployment of the spouse had a negative effect on the female sexual function.

## Introduction

Sexual function is an important aspect of women’s lives and is directly related to their sexual health and marital satisfaction (Shahhosseini et al., 2014). Sexual problems and their adverse effects on one’s sexual health are among the main health concerns in women of reproductive age (Mohammadian et al., 2019). Many women experience problems with sexual function at some point, and some have difficulties throughout their lives. Female sexual dysfunction can occur at any stage of life. It can occur at any age, in any culture, and in the context of every socioeconomic status, affecting life quality and relationship with the spouse (Du et al., 2017). In Iran, 50–75% of women of reproductive age suffer from sexual dysfunction (Alidost et al., 2021).

It is necessary to be aware of the factors influencing sexual function to perform and improve sexual function (Ricoy-Cano et al., 2020). Those who better understand these factors have more sexual assertiveness and often have the necessary ability to express their sexual needs and desires and perform better in asserting their sexual rights (Gruskin et al., 2019).

Sexual assertiveness refers to an individual’s effort to fulfill his/her rights within a specified period or to fulfill his/her inner expectations via communication with one’s sexual partner (Moyano et al., 2021). Sexual assertiveness denotes the ability to express sexual needs and related feelings and thoughts without feeling guilty or anxious and the ability to negotiate over the sexual relationship and stand on one’s rights without violating the spouse’s sexual rights (Azmoude et al., 2016). Sexual satisfaction has been higher among women with higher levels of sexual assertiveness (Zhang et al., 2022). Also, this index is an important and significant mediator in determining women’s sexual satisfaction and a predictor of higher sexual satisfaction by the sexual partner (Leclerc et al., 2015). Poor sexual assertiveness can pave the path for frequent and risky sexual relationships (McNicoll et al., 2017). Assertive resistance strategy intentions and low use of sexual refusal assertiveness have been associated with the occurrence of deterring behaviors by women, leading to humiliation, blame, hostile marital relationships, and sexual dissatisfaction (Oesterle et al., 2022). Despite the undeniable importance of sexual assertiveness in sexual quality of life, women usually face difficulties in claiming their sexual rights even if they are aware of them, leading to a type of sexual inactivity and, subsequently, to anger and compromised sexual health (Santos-Iglesias et al., 2014). Sexual assertiveness is positively related to overall mental health, satisfaction with interpersonal relationships, self-expression, self-assertion, and self-esteem (Boket et al., 2016; Hurlbert, 1991; Ménard & Offman, 2009).

Self-esteem is an important aspect of well-being and an individual’s positive or negative attitudes toward oneself (Du et al., 2017). Self-esteem is perceived as self-efficacy and a feeling of personal worth and contributes to self-confidence and self-respect (Leidger et al., 2022). One of the aspects of self-esteem influencing people’s sexual behavior is sexual self-esteem, which is a person’s emotional reaction to the mental evaluations of his/her sexual behaviors (Firoozi et al., 2016). Overall, the concept of sexual self-esteem entails a value that people assign to their sexual identity and sexual acceptability (Esmalian Khamseh et al., 2020). Studies show that high sexual self-esteem can predict a higher level of sexual satisfaction (Leidger et al., 2022; Esmalian Khamseh et al., 2020; and Beisert et al., 2020). Evaluation of sexual self-esteem among Iranian women of reproductive age indicates that many Iranian women are unaware of this aspect of sexual health and face problems such as low sexual satisfaction and increased pain during intercourse, compromising their sexual function (Farokhi et al., 2014). On the other hand, low sexual self-esteem deteriorates one’s opinion about himself/herself, satisfaction with life, ability to perceive pleasure, mutual relationships with others, and the ability to establish intimate relationships with others (Bridge et al., 2022). Also, low sexual self-esteem can be related to sexual problems, including low sexual desire (Torres-Obregon et al., 2019).

Sexual desire is an emotional state that can be activated by inner or outside signals and lead to sexual behavior (Vowels et al., 2021). It is obvious that quality fulfilling this urge has an essential role in preserving one’s and society’s health and achieving peace and comfort (Pettigrew et al., 2021). Sexual desire is the motivation to have sex and an indicator of sexual health (Torres-Obregon et al., 2019). Langeslag and Davis believe that sexual desire fluctuates over time (Langeslag et al., 2022). Gender, behavior, orientation, and sexual identity are among the factors influencing a person’s sexual desire (Mohammadian et al., 2019). Basson suggests that the wellsprings of desire in women are stirrings from feelings that are not specifically sexual. The wish for genital tension relief (or even the expectation of sexual pleasure) seems to motivate sexual receptivity in women. Furthermore, she believes that it is a woman’s level of subjective sexual arousal that is the key player in the sexual cycle, rather than overt genital arousal or internal feelings of desire and that vasocongestion and lubrication are not reliable indices of this critical ingredient (Basson, 2007). Low sexual desire not only threatens a person’s sexual health but also harms marital relationships and reduces the sexual quality of life (Bitzer et al., 2013). Today, despite an increase in our knowledge about sexual function, the nature of its mutual relationships with other sexual phenomena is less known (McNicoll et al., 2017). Besides, the mental aspects related to sexual issues vary in different societies, highlighting the importance of a better understanding of the mental aspects of sexual issues in our country in parallel with the development of educational and social interventions (Holloway et al., 2015). As noted, sexual function is an important factor that influences marital satisfaction (Shahhosseini et al., 2014). Therefore, the present research’s objective was to determine the relationship between sexual self-esteem, sexual desire, and sexual assertiveness with female sexual function.

## Method

### Research design, study context, and participants

This cross-sectional descriptive-analytical study was conducted with the participation of 592 married women of reproductive age referring to comprehensive health centers in Rasht City.

### Procedure

This study was approved by the ethics committee of Guilan University of Medical Sciences. Sample recruitment was conducted using a multi-step clustering method in a randomized manner. First, 16 comprehensive health centers affiliated with the Guilan University of Medical Sciences were classified into 4 geographical clusters: north, south, east, and west. Using a table of random numbers, 2 centers in each district (a total of 8 centers) were selected. Inside each center, married women of reproductive age, who were eligible according to our inclusion criteria, were randomly selected.

After receiving an ethical approval code from the ethics committee of Guilan University of Medical Sciences and the necessary permits from the authorities of the selected comprehensive health centers, the researcher referred to these centers. After providing full explanations regarding the objectives, protocol, and necessity of the research to participants, the checklist of the inclusion criteria was completed for all of them, and those fulfilling the inclusion criteria and willing to participate were requested to sign written informed consent.

The inclusion criteria in this study were an age of 15–49 years; giving consent for participation in the study; Iranian nationality; being able to understand the Persian language; not being in menopause; permanent wedlock; being sexually active; not having alcohol dependence (consumption of more than twice a week); not consuming any narcotics within the past 6 months; not having a physical disability; not consuming drugs for psychiatric disorders; not being pregnant; not being in the first 3 months of postpartum; not suffering from chronic diseases such as diabetes, hypertension, or other physical and mental disorders affecting sexual function; and not experiencing severe psychological distress such as recovering from an accident or the demise of a first-degree family member during the past 3 months. Also, people undergoing infertility treatment (due to the possibility of affecting sexual self-esteem) and those with a history of untreated sexual disorders were not enrolled in the study. The exclusion criterion was incomplete in responding to the questions of the questionnaire.

### Instruments

#### Structure of the questionnaire

After recording the demographic characteristics, the Halbert Index of Sexual Assertiveness, the Persian version of the short form of the Women’s Sexual Self-esteem Questionnaire, the Halbert Index of Sexual Desire, and the Female Sexual Function Index were completed by interviews. Halbert Questionnaire of Sexual Assertiveness was designed by David Farley Halbert and contains 25 questions. This questionnaire assesses a person’s ability to express his/her sexual needs and desires and is scored based on a 5-point Likert scale from always (0) to never (4). Questions 1, 3, 5, 7, 8, 9, 10, 12, 13, 17, 18, 19, and 20 are scored in a reverse manner (always = 4, never = 0). After assigning and summing up the scores, the person’s sexual assertiveness is calculated. The final score ranges from 0 to 100, with a higher score indicating higher sexual assertiveness [27]. Qeisari and Karimian have reported the questionnaire’s reliability as 0.83, validity as 0.86, and content validity as 0.91.

Women’s sexual self-esteem questionnaire (short-form) was designed by Doyle Zeanah & Schwarz (1996) and validated by Farkhi and Shareh (2014) in Iran. This questionnaire contains 32 items and 5 subscales: experience and skill (evaluating the subject’s ability to reach or give orgasm to the sexual partner and opportunities to become involved in sexual activity; items 4, 5, 13, 16, 17, and 19), sexual attractiveness (the subject’s feelings about his/her sexual attractiveness regardless of others’ opinions; items 2, 7, 12, 22, 27, 32, and 34), control (the subject’s ability to guide or manage his/her sexual thoughts, feelings, and interactions, items 6, 9, 11, 14, 21, and 29), compatibility (the harmony of a person’s sexual experiences or behaviors with his/her other personal goals or desires; items 8, 15, 23, 24, 26, and 28), and moral judgment (the harmony of a subject’s sexual thoughts, feelings, and behaviors with his/her ethical virtues; items 10, 18, 20, 25, 30, 31, and 35). The tool is scored based on a 6-point Likert scale from 1 (complete disagreement) to 6 (complete agreement). The scoring of items 6, 7, 11, 13, 14, 17, 18, 20, 21, 22, 23, 26, 27, 28, 29, 31, and 34 is in a reverse manner. The final score ranges between 32 and 192 and is calculated by summing up the scores of the 5 subscales. A higher score indicates higher sexual self-esteem. The test–retest reliability coefficient was 0.91 for the whole scale and ranged from 0.82 to 0.94 for the 5 subscales (Doyle Zeanah & Schwarz, 1996).

The Hurlbert Index of Sexual Desire (HISD; Apt & Hurlbert, 1992) was used as a measure of sexual desire. Total scores on this 25-item measure range from 0 to 100, with items rated on a 5-point Likert-type scale, ranging from 0 (all of the time) to 4 (never). Higher scores indicate higher levels of sexual desire. Scores below 50 indicated a low sexual desire, 51 to 75 moderate desire, and 75 to 100 indicated desirable. The test–retest reliability of the questionnaire was reported as 0.86 by Halbert, 1993. The internal validity of the questionnaire was reported as 0.89 by Yousefi et al., 2013. The Cronbach’s alpha in this study was calculated as 0.90.

The Female Sexual Function Index (FSFI) was developed by Rozen et al. in 2000 and contains 19 questions and 6 subscales: desire, arousal, lubrication, orgasm, pain, and satisfaction. This tool evaluates female sexual function within the past 4 weeks. The questions of this tool are scored from 0 to 5. According to the instructions provided by the developer, the final score in each dimension is calculated by summing the scores of the questions of each field multiplied by a factor value. The scores assigned are 1–5 for the sexual desire and sexual satisfaction dimensions and 0–5 for the arousal, lubrication, orgasm, and pain dimensions. The total score of the scale is obtained by summing up the scores of these 6 dimensions. According to this scale, a score less than 1 standard deviation or a total score equal to or below 26.55 is regarded as sexual dysfunction. The validity of the Persian version of this questionnaire has been approved by Mohammadi et al. (2008). The tool’s internal consistency was confirmed by Cronbach’s alpha coefficient, and the intra-class correlation coefficient (ICC) was used to determine the correlation between the scores obtained during 2 assessments, retrieving the values of 0.70 and 0.997, respectively (Mohammady et al., 2008).

### Data analysis

In this study, the test–retest method was used to determine the reliability of the questionnaires, resulting in a reliability coefficient of 98% for the sexual assertiveness questionnaire, 96% for the women’s sexual self-esteem questionnaire, 89% for the sexual desire questionnaire, and 92% for the Female Sexual Function Index. Cronbach’s alpha coefficient was used to determine the internal consistency of the queries. After completing the questionnaires, the data were entered into the SPSS software version 16. In order to describe qualitative variables, frequency distribution (percentage) was used, and quantitative variables were expressed using mean standard deviation, minimum, maximum, and 95% confidence interval. Also, the distribution of the scores of sexual assertiveness, sexual desire, and sexual self-esteem, as well as the scores of Sexual function fields, was assessed using the Kolmogorov–Smirnov test, revealing that none of these variables had a normal distribution. Therefore, the correlation of sexual function and its dimensions with sexual assertiveness, sexual desire, and sexual self-esteem was scrutinized using Spearman’s correlation. The relationship of sexual function fields with sexual assertiveness, sexual desire, and sexual self-esteem was evaluated using a multiple linear regression model by adjusting the impacts of sociodemographic and confounding variables. The statistical significance level for the tests was considered *p* < 0.05.

## Results

The minimum and maximum ages of the participants were 18 and 41 years, respectively, with a mean of 28.14 ± 3.06 years. The mean age at the time of marriage was 24.14 ± 3.25 years, and the mean age of the spouse was 29.16 ± 6.21. The average number of household members was 1.38 ± 0.3. Most of the 592 women studied had university education (54.56%) and were housewives (49.49%). Also, 6 (1.01%) of the participants were smokers, and most of them (67.22%) reported a moderate economic status (Table 1).

**Table 1:** Sociodemographic characteristics of participants and their spouses

Variables	Mean (SD) or number (percent)	
**Women’s age (years)**	28.14 ± 3.06	
**Spouse’s age (years)**	29.16 ± 6.21	
**Woman’s education level**	No formal education	2 (0.33)
	Primary/secondary	17 (2.87)
	Diploma	250 (42.42)
	University	323 (54.56)
**Spouse’s education level**	No formal education	39 (6.58)
	Primary/secondary	288 (48.64)
	Diploma	231 (39.02)
	University	34 (5.74)
**Employment status of woman**	Employment	299(50.51)
	Unemployed or housewife	293(49.49)
**Employment status of spouse**	Employment	535(90.38)
	Unemployed	57(9.62)
**Family income**	Weak	26 (4.39)
	Moderate	398 (67.22)
	Good	168 (28.37)
**Substance use**	No	580(97.97)
	Cigarettes	6 (1.01)
	Opium	1(0.16)
	Alcohol	8(1.35)
	Others	1(0.16)

*SD* standard deviation

The means ± standard deviations of the scores of sexual assertiveness, sexual desire, and sexual self-esteem scores were 56.79 ± 18.24, 49.12 ± 26.04, and 98.52 ± 6.11, respectively. The highest and lowest sexual self-esteem scores were related to the experience/skill (16.21 ± 2.4) and moral judgment (19.12 ± 1.31) dimensions, respectively.

Sexual assertiveness showed a significant positive correlation with the total score of sexual function, as well as the scores of its subscales, and the strongest and weakest correlations were related to sexual desire and sexual satisfaction domains, respectively. The participants’ sexual self-esteem also correlated significantly and positively with the total score of sexual function and the scores of all dimensions. The strongest and weakest correlations of sexual self-esteem were related to orgasm and sexual arousal, respectively. Also, there was a statistically significant correlation between the score of sexual function and all areas of sexual self-esteem except for the moral judgment domain, with the strongest correlation being related to the compatibility area and the weakest correlation to the moral judgment area (Table 2).

**Table 2:** Correlations between sexual assertiveness, sexual desire, and sexual self-esteem scores and FSFI scores

Variables			FSFI						
			**Desire**	**Arousal**	**Lubrication**	**Orgasm**	**Satisfaction**	**Pain**	**Total score**
**Sexual assertiveness**		Correlation coefficient^a^	0.369	0.263	0.191	0.258	0.207	0.115	0.13
		*p* value	0.112	< 0.01	< 0.01	< 0.01	< 0.01	< 0.01	< 0.01
**Sexual desire**		Correlation coefficient^a^	0.086	0.224	0.180	0.382	0.271	0.49	0.178
		*p* value	0.42	< 0.001	< 0.001	< 0.001	< 0.001	< 0.001	< 0.001
**Sexual self-esteem**	Experience and skill	Correlation coefficient^a^	0.36	0.215	0.215	0.151	0.407	0.246	0.207
		*p* value	0.275	< 0.001	< 0.001	< 0.001	< 0.001	< 0.001	< 0.001
	Attractiveness	Correlation coefficient^a^	0.024	0.173	0.197	0.232	0.117	0.312	0.193
		*p* value	0.21	0.035	0.0115	< 0.001	0.011	< 0.001	< 0.001
	Control	Correlation coefficient^a^	0.012	0.096	0.136	0.218	0.12	0.097	0.26
		*p* value	0.567	0.022	0.124	< 0.001	0.023	< 0.001	< 0.001
	Adaptability	Correlation coefficient^a^	0.024	0.116	0.067	0.149	0.145	0.194	0.412
		*p* value	0.161	0.052	0.108	< 0.001	0.011	< 0.001	< 0.001
	Ethical judgment	Correlation coefficient^a^	0.056	0.265	0.198	0.466	0.306	0.147	0.08
		*p* value	0.52	< 0.001	< 0.001	< 0.001	< 0.001	< 0.001	< 0.001
	Total score	Correlation coefficient^a^	0.132	0.103	0.128	0.311	0.176	0.215	0.34
		*p* value	0.13	< 0.001	< 0.001	< 0.001	< 0.001	< 0.001	< 0.001

*FSFI* Female Sexual Function Index

^a^Spearman correlation coefficient

According to the predicted regression coefficients retrieved by a multiple linear regression model, sexual assertiveness, sexual desire, and the compatibility area of the sexual self-esteem dimension were identified as predictors of the sexual function of women of reproductive age. A one-point increase in the score of the compatibility area raised the score of female sexual function by *β* ± SE = 0.079 ± 0.036 on average. All sociodemographic and confounding variables with *p* < 0.1 in the primary model entered into the final model to adjust and remove the impact of these variables on the main outcome. According to the adjusted multiple linear regression model, sexual function was significantly associated with the spouse’s job (*β* =  − 0.415, *p* = 0.003) and the family income (*β* = 0.173, *p* = 0.037). In this regard, women whose husbands were employed and had a better economic status obtained a higher sexual function score. On the other hand, the unemployment of the spouse had a negative impact on female sexual function (Table 3).

**Table 3:** Multiple regression analysis to predict FSFI score based on sociodemographic, sexual assertiveness, sexual desire, and sexual self-esteem variables

Variable	Beta	SE	*p* value	CE	
				**Low**	**High**
**Fixed model**	1.27	0.382	0.091	− 0.063	2.146
**Sexual assertiveness**	0.272	0.055	< 0.001	0.126	0.493
**Sexual desire**	0.164	0.201	< 0.001	0.173	0.294
**Sexual self-esteem (adaptability)**	0.079	0.036	0.048	0.028	0.063
**Unemployment of spouse**	− 0.415	0.258	0.003	− 1.014	− 0.159
**Family income (good)**	0.173	0.042	0.037	0.002	0.179

*SE* standard error, *CE* coefficient of error

## Discussion

The present study is among the first studies evaluating the association of sexual assertiveness, sexual desire, and sexual self-esteem with Iranian female sexual function. The mean score of sexual assertiveness was obtained at 56.79 ± 18.24. According to the results of the Spearman correlation, there was a direct and significant relationship between sexual assertiveness and female sexual function. In other words, women with a higher capability of fulfilling their sexual rights had a better sexual function as well. This finding agrees with the results of Hurlbert (1991) who showed that women with higher sexual assertiveness had better sexual function in terms of orgasm, sexual desire, sexual satisfaction, and the frequency of intercourse. Sexual assertiveness represents the ability to express true emotions and establish one’s rights by taking others’ rights into account (Topper et al., 2010). Therefore, it brings better sexual satisfaction for the couple.

Besides, the sexual assertiveness index is one of the determinants of sexual desire (Sayyadi et al., 2019). According to Brassard et al. (2015), although women’s levels of perception and awareness can affect their sexual assertiveness, even an aware woman may face difficulties in claiming her sexual rights. Incapability in asserting one’s sexual rights and women’s perception of sexual inactivity can lead to sexual dysfunction (Azmoude et al., 2016). Various sorts of sexual dysfunction can be observed among women with low sexual assertiveness, the most common of which is low sexual desire (Oesterle et al., 2022).

In this study, the mean score of sexual desire was obtained at 49.12 ± 26.04. A direct and significant relationship was observed between women’s sexual desire and sexual function. Atatki et al. (2021) showed that sexual desire plays an important role in regulating female sexual function. Hypoactive sexual desire disorder is the most common sexual problem among women (Pettigrew et al., 2021). Although multiple physical, psychological, and cognitive parameters have been identified to explain this disorder, sexual desire is one of the most important predictors of sexual function, and women with stronger sexual desire have shown a higher sexual function index (Rosen et al., 2000). The results of the present research showed that sexual self-esteem was directly and significantly related to sexual function. Women with higher sexual self-esteem better respect their sexual identity, beauty, and attractiveness as a sexual entity, and since these individuals can better manage their sexual thoughts and behaviors and adapt themselves to their spouses’ sexual preferences, they perform better sexually. This finding was consistent with the report of Brassard et al. (2015). Also, they demonstrated that there was a direct correlation between sexual function and a person’s perception of himself/herself as a sexual partner. Therefore, people who have high sexual self-esteem experience less fear and anxiety during intercourse and experience more acceptable sexual functions. Such women are able to initiate intercourse and direct the sexual relationship toward their objectives. On the other hand, women with low sexual self-esteem focus more on their spouses’ sexual satisfaction, tend not to be engaged in taking about intercourse, and rarely initiate sexual activity.

In this study, a significant and positive relationship exists between family incomes and female sexual function, which is supported by the results of Ahmadi et al. (2022) who declared that the participants with a better economic status had a higher sexual function score. One’s mind not being occupied with economic hardships, as well as peace of mind, remarkably reduces sexual dysfunction in all stages of sexual activity. Moreover, women with a better economic status have more access to health and medical counseling services. A significant negative correlation was observed between unemployment of spouse and female sexual function. In fact, the spouse’s being unemployed adversely affected the sexual function of the participants. Similarly, Saberi et al. (2018) reported a statistically significant inverse relationship between sexual function and the variables of job and income.

### Implications

The findings of the current study help clarify the relationship between sexual assertiveness, sexual desire, and sexual self-esteem with sexual function among women. These findings provide healthcare professionals with practical implications in the scope of clinical practice. With this knowledge, clinicians can explore issues related to sexual schemas that could impede sexual function. Furthermore, healthcare providers can help clients address the underlying concerns within their relationship through techniques geared toward improving sexual desire and sexual self-esteem.

### Limitation

Our research might have the following limitations: The sample was composed of married women of reproductive age. To examine the generalizability of findings, it would be useful for future studies to include a more ethnically/racially and sexually diverse sample, as well as community samples. This study used self-report measures and may be subject to social desirability. However, it is important to note that in the assessment of sexual behavior, anonymous self-administered surveys have been shown to increase rates of disclosure.

This study utilized a cross-sectional, correlational design; thus, the temporal ordering of the variables explored in this study remains unclear. Longitudinal studies are needed to confirm that changes in sexual self-esteem, sexual desire, and sexual assertiveness precede sexual function.

One of the noteworthy limitations of this study is its cross-sectional design. In addition, as sexual function involves two people who may have their levels of sexual assertiveness, sexual desire, and sexual self-esteem, future studies should focus on evaluating the sexual assertiveness, sexual desire, and sexual self-esteem of both partners and not just focus on women. The present study findings mostly show the complexity of the Iranian context for the development of the relationship between sexual assertiveness, sexual desire, sexual self-esteem, and sexual function in women; further studies that include a broader range of cultures would be advantageous, as this would improve our understanding of the impact that different cultures have on these factors.

## Conclusions

Based on the findings, sexual assertiveness, sexual desire, the adaptability of sexual self-esteem, and family income have a direct and statistically significant relationship with female sexual function.

Given there is a relationship between sexual assertiveness and sexual function, therapists should have adequate knowledge about interventions aimed at increasing sexual assertiveness. Interventions aimed at increasing sexual assertiveness before sexual activity occurs could also decrease ambiguity in the sexual context and ensure that individuals get what they want.

## Data Availability

The data that support the findings of this study are available on request from the corresponding author. The data are not publicly available due to privacy or ethical restrictions.

## Abbreviations

FSFI: Female Sexual Function Index
SD: Standard deviation
ICC: Intra-class correlation coefficient
